# The Effects of Exercise on Expression of CYP19 and StAR mRNA in Steroid-Induced Polycystic Ovaries of Female Rats 

**DOI:** 10.22074/ijfs.2018.5035

**Published:** 2017-10-14

**Authors:** Fatemeh Aghaie, Homayoun Khazali, Mehdi Hedayati, Ali Akbarnejad

**Affiliations:** 1 Department of Physiology, Faculty of Life Science and Biotechnology, Shahid Beheshti University, Tehran, Iran; 2 Cellular and Molecular Research Center, Research Institute for Endocrine Sciences, Shahid Beheshti University of Medical Sciences, Tehran, Iran; 3 College of Physical Education and Sports Sciences, University of Tehran, Tehran, Iran

**Keywords:** Cytochrome P450 Family 19, Estradiol Valerate, Exercise, Polycystic Ovarian Syndrome, Steroidogenic Acute Regulatory Protein

## Abstract

**Background:**

Polycystic ovarian syndrome (PCOS) is the most frequent female endocrine disorder that affects 5-10% of women. PCOS is characterized by hyperandrogenism, oligo-/anovulation, and polycystic ovaries. The aim of the present research is to evaluate the expression of steroidogenic acute regulatory protein (StAR) and aromatase (CYP19) mRNA in the ovaries of an estradiol valerate (EV)-induced PCOS rat model, and the effect of treadmill and running wheel (voluntary) exercise on these parameters.

**Materials and Methods:**

In this experimental study, we divided adult female Wistar rats that weighed approximately 220 ± 20 g initially into control (n=10) and PCOS (n=30). Subsequently, PCOS group were divided to PCOS, PCOS with treadmill exercise (P-ExT), and PCOS with running wheel exercise (P-ExR) groups (n=10 per group). The expressions of StAR and CYP19 mRNA in the ovaries were determined by quantitative real-time reverse transcriptase polymerase chain reaction (qRT-PCR). Data were analyzed by one-way ANOVA using SPSS software, version 16. The data were assessed at α=0.05.

**Results:**

There was significantly lower mRNA expression of CYP19 in the EV-induced PCOS, running wheel and treadmill exercise rats compared to the control group (P<0.001). Treadmill exercise (P=0.972) and running wheel exercise (P=0.839) had no significant effects on CYP19 mRNA expression compared to the PCOS group. mRNA expression of StAR in the ovaries of the PCOS group indicated an increasing trend compared to the control group, however this was not statistically significant (P=0.810). We observed that 8 weeks of running wheel and treadmill exercises could not statistically decrease StAR mRNA expression compared to the PCOS group (P=0.632).

**Conclusion:**

EV-induced PCOS in rats decreased CYP19 mRNA expression, but had no effect on StAR mRNA expression. We demonstrated that running wheel and moderate treadmill exercise could not modify CYP19 and StAR mRNA expressions.

## Introduction

Polycystic ovarian syndrome (PCOS) is a common endocrine disorder in premenopausal women characterized by ovulatory dysfunction, clinical and/or biochemical signs of hyperandrogenism, and polycystic ovarian morphology (1, 2). The clinical manifestations of PCOS include menstrual abnormalities, hirsutism, acne, and anovulatory infertility (3, 4). Although PCOS is a prevalent disease among reproductive-aged women, the etiology of this disorder remains elusive. The PCOS ovaries are enlarged bilaterally. Follicular development arrests at the stage where the selection of the dominant follicle should normally happen. As a result, a large number of small antral follicles (4-7 mm diameter) gather in the ovaries of PCOS women; rarely, a dominant follicle presents for ovulation. The mechanism responsible for arrested follicular growth in PCOS is undiscovered (1, 5). Several causes have been attributed to this defect. The majority of evidence points to the high plasma free testosterone levels and low levels of estradiol in PCOS ovaries, which appears to result from dysregulation of steroidogenesis (6). Estradiol concentrations were low in small antral follicles and began to increase in some of the 7 mm follicles of control women (7). A healthy follicle 8 mm in diameter efficiently converts androstenedione to estradiol. Conversely, atretic and/or cystic follicles have a high androstenedione to estradiol ratio. Aromatization of androgen to estradiol in dominant follicles is conducted in the granulosa cells (GC) by aromatase enzyme (8, 9).

Aromatase (CYP19) is a key steroidogenic enzyme that separately catalyzes the conversion of testosterone and androstenedione to estradiol and estrone. Aromatase is encoded by the CYP19 gene located on the long arm of chromosome 15 at position 15q21.1. Presumably, the increase in P450arom expression and estradiol production occurs in follicles that would have become large dominant follicles. Some investigations indicate that the gene which codes for CYP19 can be included as a major determinant of risk for PCOS (10). It has been assumed that GCs obtained from medium-sized follicles of women with PCOS generate very little estrogen. Therefore, estradiol production is low in PCOS follicles because of the lack of mature follicle development that produces a large amount of estradiol to increase P450arom mRNA expression (11, 12).

Hyperandrogenism is the central feature of PCOS. A chronic excessive androgen of ovarian and/or adrenal origin can result from a widespread biochemical feature such as hyperinsulinemia and hyperandrogenism in PCOS women. Intra-ovarian hyperandrogenism may be causatively linked with anovulation in PCOS. Some or all patients with PCOS may have an intrinsic, and possibly primary, abnormality of androgen biosynthesis (3, 4). One feasible cause for such an abnormality is the production of the steroidogenic acute regulatory protein (StAR). StAR mRNA has first been detected in mouse embryos and in the adrenal cortex, ovaries, testis, and kidneys (13, 14). This gene encodes StAR that plays a pivotal role by raising the conversion of cholesterol into pregnenolone. This initiates the process of steroidogenesis by facilitating the delivery of cholesterol from the outer mitochondrial membranes to the inner mitochondrial membranes of the cell. StAR has a crucial role in the regulation of steroidogenesis (15).

Physiological changes in the synthesis of steroid hormones are closely linked with alterations in StAR expression. It has been demonstrated in the ovary that StAR expression highly correlates with steroidogenic activity, because the increased production or concentration of StAR may result in abnormal steroidogenesis found in PCOS (16). Pharmacological intervention and lifestyle modification are considered treatments for PCOS and normalize hyperandrogenism and anovulation. Lifestyle intervention in PCOS includes dietary intervention and physical activity (17, 18). The most preferred, effective treatment of PCOS is physical activity. It is a key component of any lifestyle modification. Moderate regular exercise undoubtedly affects fertility and assisted reproductive technology (ART) outcomes (19, 20). Physical activity and exercise lead to fluctuation in hormone levels. Numerous evidence shows that exercise can modify body weight, improved ovulatory function, circulating androgen levels, and insulin sensitivity in women with PCOS (21). There are no studies, to our knowledge, that have assessed the effects of 8 weeks of moderate intensity (28 m/minute, 0% grade) treadmill exercise and running wheel exercise (voluntary) on StAR and CYP19 mRNA expressions in rats with PCOS. In order to test this hypothesis, we examined the ovaries from rats with PCOS to evaluate the effects of exercise on weight changes and expression of relative concentrations of StAR and CYP19 mRNA in the estradiol valerate (EV)-induced PCOS rat model.

## Materials and Methods

### Animals and protocols

In this experimental study used 40 female Wistar rats (220-240 g) acquired from Shahid Beheshti University (Iran). The rats were kept at the animal house under standard laboratory conditions (12 hour light/12 hour dark cycles and controlled temperature of 21-22ºC and 55-65% relative humidity with free access to food pellets and tap water. In order to conduct a comparative evaluation, we divided the rats into two group of 10 animals per group: control group which not receive any injection (n=10) or other manipulations and an experimental PCOS group (n=30) that received one injection of 4 mg/kg body weight EV dissolved in 0.2 ml olive oil. We recorded the weight and fertility for all of the rats. At 60 days past the EV injection, rats in PCOS group were randomly divided into three groups of 10 animals per group: PCOS (n=10), PCOS plus exercise on a treadmill (P-ExT, n=10) and PCOS plus exercise on a running wheel (P-ExR, n=10). The treatment lasted for 8 weeks. Rats in the control and PCOS groups did not participate in any exercise program. At 8 weeks after the intervention, we measured their estrous cycles daily for 21 consecutive days. The Ethics Committee at the Neuroscience Research Center of Shahid Beheshti University of Medical Sciences (Tehran, Iran) approved the study procedures.

### Physical exercise

#### Chronic voluntary exercise (running wheel)

The P-ExR rats were placed in the wheel running device for 4 hours/day with free access to water and no food. This exercise was voluntary in nature compared to the forced treadmill exercise. Running wheel distance was documented daily and we measured their body weights two times per week. This group included 10 rats that ran a total distance of 1200 m within a period of 8 weeks.

#### Physical exercise on a treadmill

The P-ExT rats were placed on the motorized treadmill for 5 days to walk for 10 minutes at 5 m/minute for training purposes. The treadmill training was performed between 8:00 and 12:00 daily. We did not change the grid during exercise. The training group was given exercise training for five days/week for 8 weeks. Each session began with 12 m/minute for 5 minutes to prepare the rats for the main training session. In the first week, the rats were trained on the treadmill at 10 m/minute, with a running time of 10 minutes/day. In the second week, we increased the speed to 15 m/minutes for 30 minutes/day. In the third week, the speed was increased to 20 m/minutes for 45 minutes/day. In the fourth week, the speed was increased at 28 m/minutes and increased the duration to 60 minutes/ day (Table 1). For the last 4 weeks, we kept the speed and duration constant. This condition corresponded to a moderate intensity of approximately 65-70% maximal oxygen consumption (22).

**Table 1:** The treadmill exercise program


Week	Duration (minutes)	Speed (m/minute)

1	10	10
2	30	15
3	45	20
4	60	28
5	60	28
6	60	28
7	60	28
8	60	28


### Quantitative real-time reverse transcriptase polymerase chain reaction

After anesthesia, we immediately removed the rats’ ovaries and froze them in liquid nitrogen. We used 5 ovaries from each group for quantitative real-time reverse transcriptase polymerase chain reaction (qRTPCR). The tissue samples were stored at -80°C until RNA extraction.

### RNA extraction

Total RNA was extracted using pureZol RNA isolation reagent according to the manufacturer’s instructions (Bio-rad, USA). The quantity and purity of RNA were measured by a nanodrop spectrophotometer (NanoDrop- 1000, Thermo Scientific, USA) with an OD260 to calculate the concentration and 260/280 to assess sample purity.

### cDNA synthesis

We determined that 5 μg of RNA was the final concentration needed to synthesis cDNA. If the concentration of RNA was 2000 ng in 1 μl, we used 2.5 μl RNA for the 5 μg RNA concentration. After DNase treatment, 5 μg of total RNA was reversed to cDNA by RevertAid reverse transcriptase (M-MuLV RT, 1 μL), random hexamer primers (1 μl), dNTPs (2 μL), and RiboLock RNase-inhibitor (0.25 μL) for 10 minutes at 25°C, followed by 60 minutes at 42°C in a final volume of 20 μL. The reaction was terminated by heating at 70°C for 5 minutes.

### Real-time polymerase chain reaction

We designed the real-time PCR primers according to the primer-BLAST tool (Table 2). The primers were synthesized by Bioneer Company (Republic of Korea). Real-time PCR was performed in duplicate for each sample on a Corbett Rotor-Gene 6000 (Adelaide, Australia). In each reaction, 1 μL of cDNA, 0.4 μL of forward and reverse primers, and 7.5 μL of SYBR Green PCR Master Mix 2X (Ampliqon, Denmark) were added with RNase-free water to make a final volume of 20 μL. All primers were used at an optimized concentration of 50 μM. Real-time PCR was performed in three steps: i. Initial denaturation (10 minutes at 95°C); ii. A three-step quantification and amplification program (15 seconds at 95°C followed by 20 seconds at 56°C and 40 seconds at 72°C) for 40 cycles; and iii. Melting curve (5 minutes at 72°C). Reactions with no template were included as negative controls, which showed no evidence of product amplification or primer dimers. The specificity of the real-time PCR reactions was verified by the generation of a melting curve analysis. The target genes were normalized with the house-keeping gene glyceraldehyde-3-phosphate dehydrogenase (*GAPDH*). The relative mRNA level of each target gene was calculated by the 2^-ΔΔCT^ method, where ΔCT (cycle threshold)=CT of target gene-CT of housekeeping and ΔΔCT= ΔCT of the target gene in the FH group-ΔCT of the target gene in the control group.

### Statistical analysis

Data were expressed as mean ± SD. All the data were analyzed using one-way ANOVA followed by post hoc Tukey’s test using the SPSS software (version 16). In all cases, P<0.05 was considered significant.

**Table 2:** Primers used for quantitative real-time polymerase chain reaction


Primer name	Primer sequence (5´→3´)	Product length (bp)	Gene bankaccession no.

CYP19	F: CGTCATGTTGCTTCTCATCGR: TACCGCAGGCTCTCGTTAAT	100	NM_017085.2
StAR	F: GCCTGAGCAAAGCGGTGTCR: CTGGCGAACTCTATCTGGGTCTGT	100	NM_031558.3
GAPDH	F: TGCCGCCTGGAGAAACCTGCR: TGAGAGCAATGCCAGCCCCA	172	NM_017008.4


## Results

### Effect of exercise on body weight

The body weight of rats from the PCOS group increased signiﬁcantly compared to the control and exercise groups. At the end of the experiment, the body weights of the exercise groups were meaningfully less compared to the PCOS group (P<0.001) (Table 3). There was a substantial effect by the EV injection on the weights of the ovaries, while there was no effect on exercise. The ovaries of both the PCOS and the PCOS exercise groups weighed less than the ovaries of the control group.

**Table 3:** Effects of the treadmill and running wheel exercises on body and ovarian weights


Variable	Control	PCOS	P-ExT	P-ExR

Body weight (g)	239.8 ± 2.48	255 ± 2.99	210.3 ± 1.87^a^	213.7 ± 1.71^a^
Ovary weight (mg)	84.5 ± 1.33	40.3 ± 1.54^b^	42.8 ± 0.9^b^	40.0 ± 1.18^b^


Significant differences are indicated by letters. Data are mean ± SEM, P<0.05, n=10 in each group. ^a^; Compared to PCOS group, ^b^; Compared to control group, PCOS; Polycystic ovarian syndrome, P-ExT; PCOS rats that exercised on a treadmill, and P-ExR; PCOS rats that exercised on a running wheel.

### Steroidogenic acute regulatory protein (StAR) and CYP`19 mRNA expressions

#### Ovarian expression of aromatase (CYP19)

**Fig.1:**
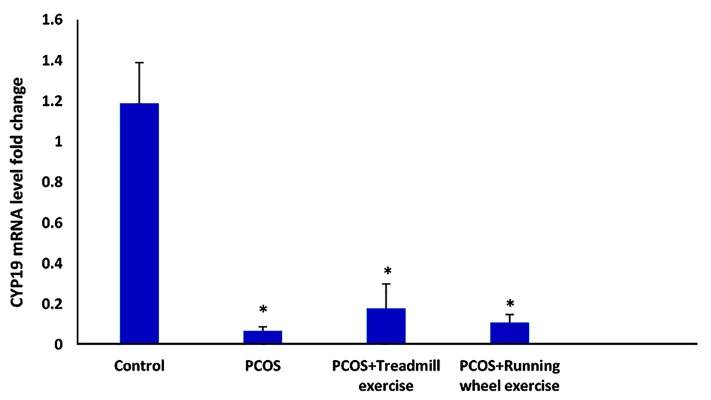
Effect of exercise (running wheel and treadmill) on the expression of ovarian aromatase (CYP19) mRNA in the polycystic ovarian syndrome (PCOS) group in estradiol valerate (EV)-induced PCOS rats. Values are mean ± SD. *; P<0.05 vs. control (Tukey post-hoc).

There was significantly lower mRNA expression of CYP19 in the EV-induced PCOS, P-ExR, and P-ExT rats compared to the control group (P<0.001). There was no significant effect of treadmill exercise (P=0.972) or running wheel exercise (P=0.839) on CYP19 mRNA expression compared to the PCOS group (Fig .1).

#### Ovarian expression of steroidogenic acute regulatory protein (StAR)

**Fig.2:**
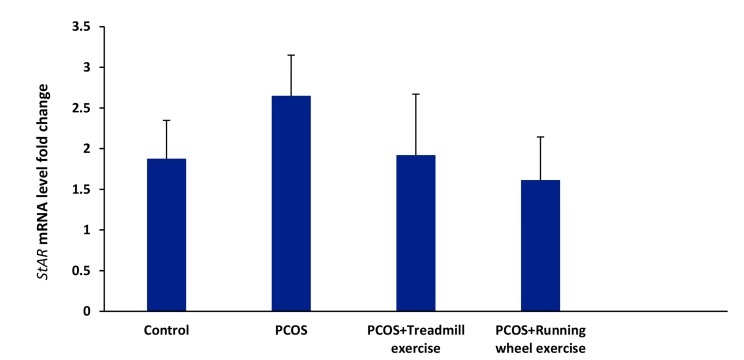
Effect of exercise (running wheel and treadmill) on the expression of ovarian steroidogenic acute regulatory protein (StAR) mRNA in the polycystic ovarian syndrome (PCOS) group in estradiol valerate (EV)-induced PCOS rats. Values are mean ± SD.

The PCOS group had a higher level of StAR mRNA expression compared to the control group although this finding was not statistically significant (P=0.810). The 8 weeks of running wheel and treadmill exercises could not statistically decrease StAR mRNA expression compared to the PCOS group (P=0.632) (Fig .2).

## Discussion

PCOS is a term for unexplained hyperandrogenism associated with variable degrees of cutaneous and anovulatory symptoms, and obesity (23, 24). In PCOS, follicular development arrests at the stage when the GCs of the growing follicles would normally begin to express the aromatase enzyme and secrete estradiol. However, several abnormalities of PCOS include a significant increase in androgens and luteinizing hormone (LH), and reduced follicle-stimulating hormone (FSH). In PCOS, hypersecretion of LH leads to excessive androgen levels. On the other hand, FSH is vital for maturing follicles through FSH receptors (FSHRs) in GC of antral follicles which contributes to aromatase transcription (26).

In PCOS, the expression of FSHR in GC appears to be up-regulated (27), which is believed to be responsible for the observed hyper-responsiveness of PCOS GC to FSH both *in vitro* and *in vivo* (28, 29). Although FSH hypersensitivity hastens estrogen production, PCOS women are unable to sustain their estrogen levels. One of the reasons for insufficient production of estradiol in PCOS follicles is the inadequate amount of aromatase to stimulate bioactivity and increase aromatase mRNA expression (28). Takayama et al. (30) reported that immunohistochemical studies of polycystic ovaries did not show any aromatase activity in antral follicles of various sizes (30). Erickso et al. (12) demonstrated that GC obtained from mediumsized follicles of women with PCOS had little aromatase activity. Similarly, Jakimiuk et al. (11) showed that when compared to the control follicles, all PCOS follicles contained low levels of P450arom mRNA estradiol and lower aromatase stimulating bioactivity.

Yang et al. (31) reported that freshly isolated GC from luteinized follicles in PCOS had significantly reduced aromatase mRNA and protein. The results of this study have supported that testosterone is a key factor responsible for down-regulation of aromatase in PCOS. At the average level in small follicles in PCOS patients, testosterone down-regulated both mRNA and protein levels of aromatase in cultured non-PCOS luteinized GC (31). Consistent with previous studies, this research demonstrated that decreased levels of P450arom mRNA in PCOS rats compared to the control group. The 8 weeks of running wheel and treadmill exercises could not increase CYP19 mRNA expression. When LH affects its receptor on the theca cell, StAR initiates the process of steroidogenesis by transporting cholesterol from the outer to the inner mitochondrial membrane (32, 33).

StAR has a crucial role in binding to cholesterol. It is the first and rate limiting step of steroidogenesis, hence, it has been considered a major candidate for high levels of steroid hormones in PCOS patients (14, 34). Jakubowski (35) reported that StAR was one of the candidate genes involved in PCOS. Kahsar-Miller et al. (15) revealed that alteration in the StAR gene could cause PCOS as a reason in the earliest steps of androgen biosynthesis. They observed that the relative concentration and distribution of StAR in the PCOS ovary did not differ significantly from the normal ovary. However, they reported that the StAR protein was located primarily in the thecal and GC of follicular cysts. The StAR protein content in the entire ovary as determined by Western blot analysis tended to be greater in patients with PCOS than controls.

Wickenheisser et al. (36) demonstrated that StAR promoter activity was similar in PCOS and normal control cells. There were similar levels of StAR mRNA in both PCOS and normal theca cells. Nelson et al. (37) did not observe elevated StAR mRNA expression in PCOS compared to normal cells. Urbanek et al. (38) reported no association between PCOS or hyperandrogenemia and the StAR locus. In accordance with the previous studies, mRNA expression of StAR in the ovary of the PCOS group was not very different, though partially elevated, compared to the control group. However, the treadmill and running wheel exercises failed to significantly affect StAR mRNA expression.

Intervention studies to see if exercise can modify StAR and CYP19 mRNA expression in women with PCOS are lacking both in human and animal, as the study in animal models. There is no study about the effect of exercise on StAR and CYP19 mRNA expression in PCOS. Knowler et al. (39) have recently demonstrated that a 24-week training period that included 30 minutes bicycling three times a week in PCOS women resulted in a significant decrease in fasting insulin and insulin resistance. Vigorito et al. (40) reported that bicycling for 40 minutes 3 times a week for 3 months in obese patients with PCOS led to an improvement in insulin sensitivity. Qiu et al. (41) demonstrated that 2 weeks of swimming in the testosterone propionate-induced PCOS rat improved insulin sensitivity, decreased serum androgen levels, and recovered normal ovarian morphology. Homa et al. (42) reported that voluntary exercise improved estrous cycle in prenatallyandrogenized female mice.

## Conclusion

The current study ﬁndings have indicated that EV-induced PCOS in rats is linked to decreased CYP19 mRNA expression with no effect on StAR mRNA expression. We have determined that voluntary exercise (running wheel exercise) and moderate treadmill exercise could not alter CYP19 mRNA and StAR mRNA expressions. However, we only measured two genes; hence, future studies could survey other genes considered vital in the steroidogenesis pathway in PCOS. It seemed that exercise might affect PCOS via another pathway. This investigation has provided a new way to investigate the effect of exercise on expression of critical genes in PCOS.
